# Acid reducing agents to neonates – lack of evidence and guidelines

**DOI:** 10.3109/21556660.2012.655816

**Published:** 2012-01-09

**Authors:** Stina Paulsson, Staffan Eksborg, Åsa Andersson, Per Nydert, Lena Grahnquist

**Affiliations:** 1 Karolinska Pharmacy, Karolinska University Hospital, StockholmSweden; 2 Department of Women’s and Children’s Health, Karolinska Institutet; Karolinska Pharmacy; and Astrid Lindgren Children’s Hospital, Karolinska University Hospital, StockholmSweden; 3 Karolinska Pharmacy and Astrid Lindgren Children’s Hospital, Karolinska University Hospital, StockholmSweden; 4 Department of Women’s and Children’s Health, Karolinska Institutet and Astrid Lindgren Children’s Hospital, Karolinska University Hospital, StockholmSweden

**Keywords:** Histamine_2_ receptor antagonists, Neonates, Pharmaceutical preparations, Proton pump inhibitors

## Abstract

**Objective:**

The aim of this retrospective study was to investigate the clinical practice, i.e. the frequency of use and the treatment strategies, for acid reducing drugs to neonates in a Swedish hospital.

**Methods:**

Retrospective reviews of charts and interviews with nurses at the neonatal wards of Karolinska University Hospital were performed to identify difficulties that might occur with drug administration. All patients admitted over a 2-month period were included. Main outcome measure were the number of patients treated with acid reducing drugs and the dosages.

**Results:**

Nine out of 215 patients (4.2%) received an acid reducing drug. Patients treated with acid reducing drugs had significantly lower birth weight, lower gestational age and longer duration of hospitalization. Eight of the patients were treated with omeprazole. One of these patients started treatment with omeprazole but continued later on with ranitidine. One patient was exclusively treated with ranitidine. The doses of omeprazole (intravenous or oral administration) were within the range 0.16–1.26 mg/kg/day.

**Conclusions:**

A wide variation in treatment regimens of acid reducing drugs is given to newborn infants. The percentage of treated children was much lower than earlier reports from the US and UK. No conclusions can be drawn as to whether the doses and dosing intervals used give sufficient acid suppression, since the effect of the therapy was not recorded. The present study is only retrospective and data are not truly comparable with other studies. Further studies are therefore warranted to evaluate effective doses and pharmacokinetics of acid reducing drugs in newborn infants.

## Introduction

Indications for acid reducing drugs in the pediatric population include ulcer diseases and disorders relating to gastroesophageal reflux^1^. These diseases appear differently in children that in adults. Whereas acid reducing drugs are commonly used in otherwise healthy adults, children in need of them are commonly sicker and more vulnerable. Owing to this fact, there is a lack of studies on acid reducing drugs in sick or very young children. In neonates current knowledge regarding effect and safety of acid reducing therapy is limited.

The presence of parietal cell expression of H^+^, K^+^-ATPase and production of gastric acid in both preterm and term infants have been demonstrated in several studies^2,3^.

Gastroesophageal reflux is a common condition in premature infants. There is an increased risk for esophageal adenocarcinoma among infants born preterm and/or small for gestational age^4,5^. However, there is no evidence that gastroesophageal reflux is the underlying cause or that acid reducing therapy would reduce the risk for esophageal adenocarcinoma. Gastric mucosal damage occurs frequently in neonates in intensive care units^6^, but the clinical significance of the damage is not completely clear.

The two types of acid reducing drugs available today are histamine_2_ receptor antagonists (H_2_RAs) and proton pump inhibitors (PPIs). At the time of the study the H_2_RA ranitidine only existed in a liquid formulation, while PPIs could only be administrated as water dispersible tablets (e.g., multi-unit pellet systems) or intravenously. The pellets within the PPI capsules and tablets are designed to protect the substance from gastric acid. The nasogastric tubes used in neonatal patients are narrow, often less or equal in size to 6 French. Thus, there is an obvious risk of tube blockage when the granules of an open capsule or enteric coated microgranules are suspended in liquid and delivered through a nasogastric tube with a narrow inner diameter. Risk of tube blockage depends on dispersion volume and syringe and tube size, as well as the number and size of the multi-unit pellet systems^7^.

None of the existing acid reducing drugs are approved by the Swedish medical agency for use in children under the age of 1 year. The acid reducing drugs used in neonatal intensive care today are therefore prescribed off-label, (i.e., outside age, indication and/or route of administration approved in the Summary of Product Characteristics). Because there is only a limited number of published studies evaluating safety and effect there are no general guidelines in Sweden for acid reducing therapy in this population.

The objective of this study was to investigate the frequency of use and the treatment strategies for acid reducing drugs in neonates. The study was performed at the three neonatal units of Karolinska University Hospital in Stockholm. More than 2200 newborn infants are treated at these units each year, representing approximately 25% of Swedish neonatal patients. This study intends to contribute to an increased knowledge of how these drugs are being used in neonates since there are no guidelines available.

## Patients and methods

### Review of charts

Retrospective reviews of charts were performed at the three neonatal units. All patients admitted over a 2-months period (November and December 2004) were included in the study. Medical charts from all patients admitted at any time during these 2 months were carefully reviewed.

Information about birth weight, gestational age and duration of hospitalization was obtained from all charts. Additional information on drug, dose, route of administration, formulation, length of treatment and indications was documented for children that had been treated with acid reducing drugs. Information about sodium concentrations in serum were obtained from patients who received an omeprazole-bicarbonate solution.

The identity of included patients was protected by assigning them a non-traceable number at the time of input, and no patient identifying details were kept. The study, performed as a quality improvement project, was approved by the chief physician.

### Interviews

Interviews with nurses were performed (February and March 2005) to identify difficulties that might occur with drug administration, especially with the proton pump inhibitors. The questions were based on a previously used questionnaire focusing on the problems of preparing the drug for administration^8^.

## Results

### Retrospective review of charts

In all, 223 infants were admitted to the three neonatal wards at the Karolinska University Hospital during the 2 study months (November and December 2004). Nine out of 215 patients (4.2%; 95% CI: 1.9–7.8%) whose charts were available (eight charts were missing) had received an acid reducing drug (Table 1).

**Table 1.** Routes of administration, doses and length of treatment for patients receiving omeprazole and/or ranitidine.

Patient number	Acid reducing drug	Administration	Initial dose (mg/kg/day)	Final dose (mg/kg/day)	Days of treatment
1	Omeprazole	IV	0.93	0.73	10
1*	Ranitidine	NG	1.26	1.23	3
2	Omeprazole	IV	0.93	0.86	10
3	Omeprazole	IV	0.24	0.20	11
4	Omeprazole	IV	0.21	0.16	14
5	Omeprazole	IV	0.22	0.22	5
6	Omeprazole	IV	0.50	0.45	7
7	Omeprazole	IV	1.04	1.04	1
8	Ranitidine	NG	7.68	2.35	13
9	Omeprazole	NG	0.94	0.79	13

*Patient no. 1 started treatment with omeprazole for 10 days and then continued with ranitidine. IV, intravenous; NG, nasogastric.

### Patients

**Figure 1.**
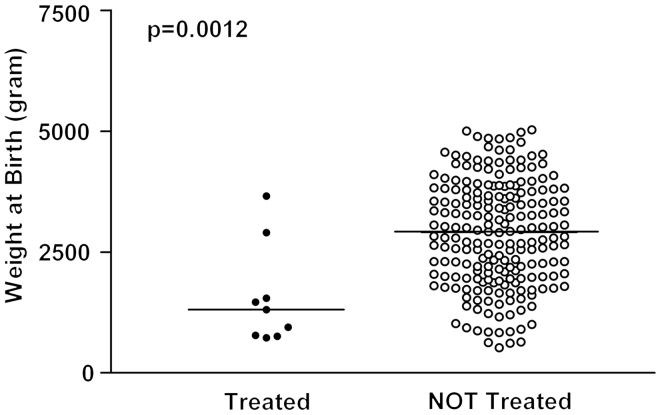
Birth weight in patients treated with acid reducing drugs versus non-treated patients. The median values are expressed by the solid lines.

**Figure 2.**
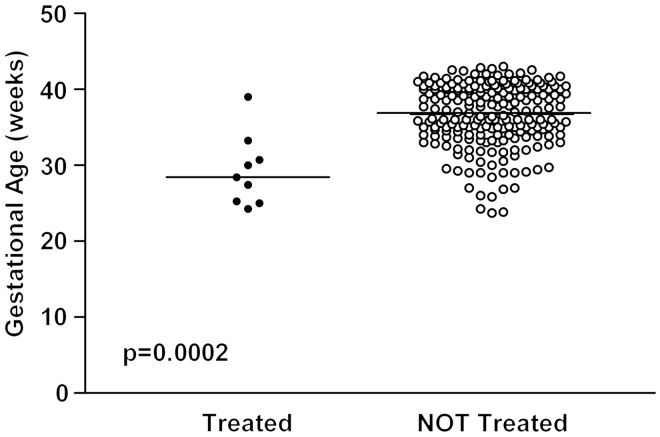
Gestational age in patients treated with acid reducing drugs versus non-treated patients. The median values are expressed by the solid lines.

**Figure 3.**
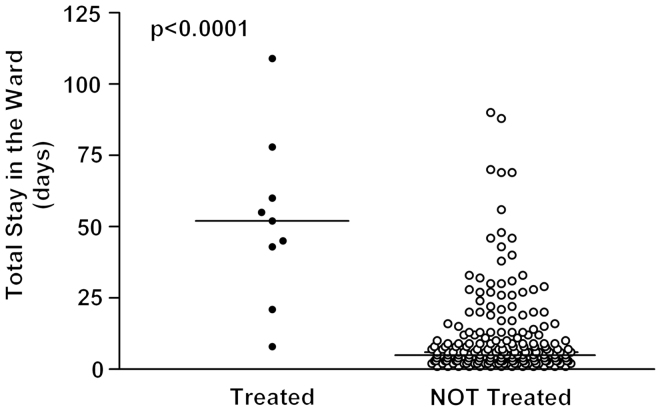
Days of hospitalization in patients treated with acid reducing drugs versus non-treated patients. The median values are expressed by the solid lines.

Patients treated with acid reducing drugs had significantly lower birth weight, lower gestational age and longer duration of hospitalization compared to the other patients in the study (Figures 1–3). A total of 111 out of 215 patients (51.6% 95% CI: 44.7–58.5%) were born before week 37, eight of whom (7.2%; 95% CI: 3.2–13.7%) received an acid reducing drug.

### Doses, treatment times and routes of administration

Eight patients were treated with omeprazole. One of these patients started treatment with omeprazole but continued with ranitidine. One patient was exclusively treated with ranitidine. The formulations used were Losec (40 mg/mL omeprazole) intravenous solutions and Zantac (15 mg/mL ranitidine) oral solution (Table 1).

Patient 9 received omeprazole intravenous solution, buffered with bicarbonate solution via a nasogastric tube. In this patient, sodium concentration in serum did not change over time, being within the range 138–142 mmol/L 10 days prior to treatment, during treatment and for 10 days post-treatment.

### Treatment strategy

The patients receiving omeprazole were on a once-daily regimen and the duration of treatment varied between 1 and 14 days (Table 2). The prescribed dose of omeprazole did not change during the treatment periods. Ranitidine was given three to four times daily during the first days of treatment (Patient 8). Thereafter the number of doses per day was reduced. Patient 1 was given ranitidine once or twice daily (Table 1). The acid reducing effect was not evaluated in any of the patients.

**Table 2.** Time for acid reducing treatment.

Patient number	Gestation age at birth (weeks + days)	Age at first dose (weeks + days)	Days of hospitalization before first dose	Days of hospitalization after last dose
1	25 + 2	28 + 5	24	6
2	24 + 2	33 + 0	61	38
3	30 + 2	33 + 5	24	10
4	30 + 5	34 + 3	26	12
5	39 + 2	39 + 5	3	4
6	27 + 3	29 + 2	13	51
7	33 + 2	33 + 4	2	17
8	28 + 3	37 + 3	42	0
9	25 + 3	32 + 1	47	22

The days of hospitalization before and after acid reducing treatment were on the average 27 and 18 days, respectively. One of the patients (Patient 8), who received ranitidine at the ward, continued treatment after discharge (Table 2).

### Indications for treatment

Indications as stated in the charts for omeprazole or ranitidine were blood in faces and/or bloody vomits (Patients 3, 4, 5, 6 and 7) or gastroesophageal reflux as a suspected cause of apnea (Patients 1, 8 and 9). No indication for treatment with acid reducing therapy was found in the chart for one of the patients (Patient 2).

### Interviews

In total, five nurses from the three neonatal units were interviewed about drug administration. Most of the interviewed nurses believed that stress ulceration was the main reason for acid reducing treatment.

The interviews confirmed that in one of the wards oral omeprazole via nasogastric tubes was preferred as acid reducing therapy. The formulation used for nasogastric administration was omeprazole solution for intravenous injection buffered with sodium bicarbonate solution. At one of the other wards, suspensions of omeprazole (i.e., microgranules) had been used for nasogastric administration. Suspensions of lanzoprazole (i.e., granules with smaller microgranules that can be dissolved and used as an oral solution) had been used for nasogastric administration at both wards.

## Discussion

Nine out of 215 patients (4.2%) received an acid reducing drug (omeprazole or ranitidine) during their stay at a neonatal ward. Doses and length of treatment varied widely in the investigated population. The neonates treated with acid reducing drugs had significant lower birth weight, lower gestational age and longer duration of hospitalization.

Indications for treatment with proton pump inhibitors or histamine_2_ receptor antagonists were gastroesophageal reflux as a suspected cause of apnea or blood in faces and/or bloody vomits. All children treated for gastroesophageal reflux disease were premature (born before week 37^9^). The patients treated with acid reducing drugs in this study were even more vulnerable and sicker than the non-treated patients reflected by gestational age, weight and length of stay (Figures 1–3).

A majority of the patients were treated by the intravenous route of administration but three of the patients were given the acid reducing drug via a nasogastric tube. The documentation for use of intravenous omeprazole and ranitidine to infants is very limited. One study including nine children between 4.5 and 27 months of age showed that an omeprazole dose of 40 mg/1.73 m^2^/day (corresponding to 1.16 mg/kg) was required to keep 24 hour gastric pH above 4 more than 90% of the time^10^.

Studies on oral administration of PPI to children are somewhat more extensive^11–17^. An effective dose of omeprazole within the range of 0.7–3.3 mg/kg/day has been identified for children 0.8–17 years of age.

One patient in our study received omeprazole solution for i.v. use (0.94 mg/kg) buffered with sodium bicarbonate once-daily administered via a nasogastric tube. The efficacy of gastric administration of an intravenous omeprazole suspension has previously been evaluated^11–13^ showing an acid reducing effect in two of the three studies. The efficacy and safety of an omeprazole formulation buffered with sodium bicarbonate have to the best of our knowledge not been adequately evaluated in neonates. Recently, a randomized double-blind placebo-controlled crossover study used the intravenous formulation of omeprazole for nasogastric administration to preterm infants after adding an antacid solution (Mylanta), showing that 0.7 mg of omeprazole/kg/day significantly reduced gastric acidity and esophageal acid exposure compared to placebo^13^.

Two patients in the present study were treated with ranitidine in markedly different doses. Prophylactic ranitidine treatment has been shown to prevent gastric mucosal lesions in newborn infants in intensive care^18^. Preterm neonates need significantly lower doses of ranitidine as compared to term neonates to keep gastric pH over 4^19^. The association between acid reducing treatment and necrotizing enterocolitis has been reported^20^. The efficacy of proton pump inhibitors and histamine_2_ receptor antagonists in children has to the best of our knowledge only been compared in one study. The included children were within the range of 6 months to 13.4 years of age. An oral dose of 40 mg/1.73 m^2^/day omeprazole was comparable to a high-dose ranitidine of 20 mg/kg/day for the healing of esophagitis and improvement of symptoms^21^.

We studied the treatment strategies using both retrospective reviews of charts and interviews with nurses. They show a wide variety and lack of coherence. We speculate that the wide variation in treatment regimes is due to the lack of general recommendations for acid reducing therapy in new born infants. The published data on randomized controlled studies are sparse and existing data are often pooled together with data on older children, hence making the few data non-specific.

The use of off-label drugs for neonates is common albeit with lack of documentation. To the best of our knowledge there are only two published studies focusing on the use of acid reducing substances in neonates. One British study, based on standard questionnaires, described a wide variation of incidence of gastroesophageal reflux diagnostic and treatment regimes at neonatal intensive care units^22^. The other study, from the United States, shows that the use of antireflux drugs in extremely low-birth-weight infants at discharge from hospitals varied widely between centers within the National Institute of Child Health and Human Development Neonatal Network. Treatment with antireflux medications in infants discharged at postmenstrual age (PMA) of ≤42 weeks varied between 1.7 and 45.7%, and for infants discharged at PMA of >42 weeks, between 22.2 and 90%^23^. The latter study also shows that the use of proton pump inhibitors in infants in the US has increased 4-fold from 2000 to 2004.

We investigated clinical practice for acid reducing drugs in newborn children at the neonatal wards of a Swedish hospital, comprising 25% of all neonatal patients in Sweden. Our study showed a lower percentage of treated children as compared to the studies from the US and UK. It is the first study in Sweden to examine clinical practice of acid reducing drugs in neonatal wards. The present study is also the only retrospective study that describes in depth the use of acid suppressive treatment in a large neonatal population. However, the data are not directly comparable with the US and UK studies.

Because of the low percentage of treated children, individual practice among the doctors could be a confounder. A larger study could have minimized this. The strength with the present study is that it has dealt with every single case in depth. The limitations are the fact that, although a large number of patients were included, only a few had been treated with acid reducing drugs. The lack of a validated treatment regime has not changed over the last decade. Reported side-effects have been debated at international forums^20^, but have not impacted on the use in our neonatal population. Today, the use of intravenous omeprazole has changed to intravenous esomeprazole and oral administration is mainly carried out with esomeprazole granules for pediatric use, and the previous wide dosing strategy has now changed to 1 mg/kg, which is described as safe, but not superior to placebo for children >1 month of age in the pharmacokinetic information supplied by AstraZeneca. We postulate that findings in the present study are still valid in Sweden. There is a lack of evidence for the need and effect of acid reducing drugs in the neonatal population. There are no guidelines for treatment with acid reducing drugs in the most vulnerable patients, i.e. the neonates. We also speculate that this could be the case with other off-label drugs in neonatal care and that the present study should encourage similar studies on other drugs.

## Conclusions

There is a wide variation in treatment regimens of acid reducing drugs given to newborn infants. The percentage of treated children are much lower than earlier reports from the US and UK. No conclusions can be drawn as to whether the doses and dosing intervals used gave sufficient acid suppression, since the effect of the therapy was not recorded. The present study is only a retrospective study and data are not truly comparable with other studies. Further studies are therefore warranted to evaluate effective doses and pharmacokinetics of acid reducing drugs in newborn infants.

## Transparency

### Declaration of financial/other relationships

None of the authors have any conflicts of interests. L.G. is a pediatrician employed by Karolinska University Hospital. S.E., Å.A. and P.N. were at the time of the study employed by the Karolinska Pharmacy.
